# Mucosal-Type Chronic Otitis Media: Clinical Predictors of Postoperative Success

**DOI:** 10.7759/cureus.56213

**Published:** 2024-03-15

**Authors:** Feba A Sunny, Rajeswari Nalamate, Sindhusha Chandran, Pradeep Kumar, Mary Kurien

**Affiliations:** 1 Otolaryngology, Pondicherry Institute of Medical Sciences, Pondicherry, IND; 2 Speech Language Sciences, All India Institute of Speech and Hearing, Mysore, IND; 3 Audiology, Pondicherry Institute of Medical Sciences, Pondicherry, IND

**Keywords:** surgical outcome, hearing outcome, graft success, clinical predictors, chronic mucosal otitis media

## Abstract

Objectives: Chronic otitis media (COM) remains a global disease, a burden, and a challenge. Predicting its treatment’s postoperative success based on clinical presentation has not been reported, particularly for the mucosal (tubotympanic/safe) type.

Methods: A prospective descriptive study of patients with mucosal-type COM was done to identify clinical predictors of improved postoperative hearing outcomes and successful graft uptakes.

Results: Among the 110 ears studied, tympanoplasty was performed in 76 and cortical mastoidectomy with tympanoplasty in 34, based on six weeks of discharge-free or persistent discharge, respectively; all were treated with postoperative intranasal steroid spray. Eustachian tube dysfunction was noted in 96.4%. All patients with a history of ear discharge over five years had ossicular discontinuity, and those with persistent discharge had nonpatent aditus. Normal postoperative hearing was attained in most patients with less than one year of ear discharge. Surgical intervention within a year achieved normal hearing and graft success of 100% with type I tympanoplasty using the fascia alone in discharge-free ears and cortical mastoidectomy with tympanoplasty using cartilage-fascia graft in discharging ears, despite Eustachian tube dysfunction. In the latter group, graft success in type II tympanoplasty was 76.9%.

Conclusion: Ear discharge is the prime predictor of postoperative success in mucosal-type COM, as its duration and persistence dictate the time and type of surgical intervention. Duration of more than five years is directly proportional to pre-operative hearing loss with ossicular involvement and inversely proportional to postoperative hearing improvement, necessitating early surgical intervention, despite it being the mucosal or “safe type.” Ear discharge-free for six weeks is an indicator of tympanoplasty. Persistent ear discharge, despite nonotogenic confounders, suggests aditus nonpatency, indicates the need for cortical mastoidectomy, and necessitates achieving its patency along with tympanoplasty. A surgical decision-making algorithm for the best possible surgical outcome in the chronic mucosal type of OM is also suggested.

## Introduction

The global burden of illness from chronic otitis media (COM) affects 65 to 330 million people; 90% is borne by developing countries in Asia, Africa, and the Western Pacific [1]. The incidence of chronic suppurative otitis media (OM) has been reported to be 4.76 per thousand (31 million cases) globally, with 22.6% of cases occurring annually in under-five-year-old children. Oceania has the highest incidence, followed by sub-Saharan and central Africa, with 9.37 and 7.56 per thousand, respectively. The global prevalence of hearing impairment caused by OM (defined as permanent hearing loss for best ear > 25 dB) was reported as 30.82/10,000, with South Asia representing 6.02/1,000 and 3.02/1,000 and 2.2/1,000 in Oceania and Africa, respectively [2].

In India, OM is the most common etiological factor (57.25%) of hearing loss, with the majority (62.2%) in the 0-20 years age group [3]. The prevalence of chronic suppurative OM among children is significantly higher in urban slums (9.9%) as compared to rural areas (7%) and nonslum urban locales (4.6%) [4].

OM’s risk factors are lack of breastfeeding, use of a pacifier or bottle feeding, poor nutritional status, exposure to smoke, allergy, over-crowding, daycare attendance, and immune deficiencies [2], as well as low social class and indoor cooking [5]. A meta-analysis also reported allergy or atopy, recurrent upper respiratory tract infection, previous history of acute or recurrent OM, and low social status as risk factors [6]. The source of infection could also be sinonasal abnormalities and allergic rhinitis [7]. Postoperative complications, diabetes mellitus, and lower air conduction thresholds were reported to be significantly independent factors [8]. Older age and the use of antithrombotic agents during hospitalization have also been reported to be independently associated with early local wound complications among those who had tympanoplasty for COM [9].

Eustachian tube (ET) function is an integral part of a normally functioning middle ear for pressure equalization and ventilation, mucociliary clearance of secretions from the middle ear, and protection against nasopharyngeal flora. A well-ventilated middle ear is also essential for successful middle ear reconstruction. Therefore, a preoperative ET function test is advised prior to tympanoplasty [10,11].

Predicting postoperative clinical success in chronic mucosal OM, based on clinical presentation, regarding both hearing improvement and graft success, has not been reported previously. This prospective descriptive study among patients with mucosal-type COM was thus undertaken with these objectives: to assess the postoperative outcome regarding graft uptake and hearing status at two and six months and to study the effect of associated clinical parameters on postoperative outcomes.

## Materials and methods

This study included consecutive patients diagnosed with COM (mucosal type) undergoing tympanoplasty with or without cortical mastoidectomy. Adults with uncontrolled hypertension and chronic kidney and cardiac diseases were excluded. This study was conducted in a tertiary academic center (Pondicherry Institute of Medical Sciences) from January 2017 to June 2018 following clearance from the Institutional Review Board and Ethics Committee (IEC: RC/16/87). Written consent was obtained from the patients/guardians prior to enrolling in the study. A detailed history was taken, and an ENT examination was performed and noted on the case report form. Pure tone audiometry and impedance audiometry with the inflation/deflation test (for ET function) were also conducted [12]. A diagnostic nasal endoscopy was done to check for any associated nasal pathologies. Blood investigations like complete blood count, serology, liver and renal function tests, random blood sugars (if diabetic fasting and postprandial sugars), including X-rays of the mastoids, were also done. An ear swab was taken to test for culture and sensitivity.

After obtaining preanesthetic clearance, patients were taken for suitable ear surgery, either tympanoplasty or tympanoplasty with cortical mastoidectomy. Those patients with a history of more than six weeks of persistent ear discharge, despite having been treated for pharyngeal or nasal pathologies and culture-specific antibiotics, underwent cortical mastoidectomy with tympanoplasty; those patients whose ears were dry underwent tympanoplasty alone. Intraoperative findings such as ossicular chain continuity, middle ear/mastoid pathology, and aditus patency were noted. All patients with dry ears were started on amoxicillin with clavulanic acid preoperatively; those patients with discharging ears were started on culture-specific antibiotics, and each was continued for 7-10 days. Mastoid dressings were changed on the second and fourth postoperative days, and the wound site was inspected. The postaural sutures were removed on the seventh postoperative day. All patients were on postoperative steroid nasal sprays for four to six weeks. Patients were reviewed two weeks after discharge and followed up for six months. Postoperative pure tone audiograms were done at two and six months.

Descriptive statistics, such as mean and standard deviation for continuous variables, numbers, and percentages for categorical variables, were given. The association between outcomes and clinical parameters was assessed using the chi-square test. The difference between the group means was measured using the t-test and ANOVA. A p-value of < 0.005 was considered significant.

## Results

There were 109 patients with 110 ears (one patient had a bilateral procedure); the female-to-male ratio was 6:5. Their ages ranged from 10 to 60 years, and most were in the 21-40 age group. Most of the patients had unilateral disease (right: 36%, left: 46%), and 92% had no nasal or throat pathology. Associated adenoid enlargement was noted in 4%, deviated nasal septum and allergic rhinitis were noted in 2% each. The majority had large central perforations (51.8%), followed by moderate-sized perforations (35.5%), with small and subtotal perforations at 8.2% and 4.5%, respectively. Forty-nine patients (45%) who had ear discharge for more than three years were found to have large or subtotal perforations, while only 13 patients (12%) who had ear discharge for less than three years had large (45%) or subtotal perforations (12%). This approached statistical significance (Table 1).

**Table 1:** Size of perforation and duration of ear discharge

Size of Perforation	Duration											Chi-Square P-value
	<1 Year			1-3 Years			3-5 Years			>5 Years		
		%			%			%			%	0.067
Small	2	6.7%	0		0.0%	0		0.0%	6		11.1%	
Moderate	9	60.0%	9		47.4%	7		31.8%	14		25.9%	
Large	4	26.7%	8		42.1%	15		68.2%	30		55.6%	
Subtotal	0	0.0%	1		5.3%	0		0.0%	4		7.4%	
Total	15	100%	19		100%	22		100%	54		100%	

Hearing loss ranged from 20 to 40 dB in 75% to > 40 dB in 25% (Table 2).

**Table 2:** Puretone average in patients dB HL: Decibel Hearing loss

Pure Tone Average	Number	Percentage
0-20 dB HL	0	0
20-40 dB HL	83	75
40-50 dB HL	16	15
>50 dB HL	11	10
Total	110	100

Analyzing the number of years of ear discharge, the mean hearing loss was 32.73 dB +/- 12.54 among those with < one year compared to 38.05 dB +/- 15.10 dB among those with > five years (Table 3).

**Table 3:** Duration of discharge with the level of hearing loss dB HL: Decibel Hearing loss, SD: standard deviation

Duration	Baseline (dB HL)		6 Months -Postoperative (dB HL)	
	Mean	SD	Mean	SD
<1 Year	32.73	12.54	25.60	10.74
1-3 Years	30.85	6.20	26.30	3.04
3-5 Years	35.59	8.32	27.41	9.81
>5 Years	38.05	15.10	30.11	11.67

When correlating the size of the perforation with hearing loss, it was noted that in those with large and subtotal perforations, mean hearing loss was 38 dB and 40 dB, respectively; those with small or medium perforations had mean hearing loss of 26 dB and 28 dB, respectively. There was impedance audiometric evidence of ET dysfunction in 96.4% of patients.

Intraoperatively, the malleus was partially eroded or foreshortened in 19%, and ossicular discontinuity with erosion of the lenticular process of incus was noted in 11.8%. The stapes superstructure was present in all. Among those with eroded incus, 90% had moderate to large perforations, and 75% had > five years of ear discharge.

Type I tympanoplasty with temporalis fascia was done in 69% of patients, while 31% had cortical mastoidectomy. Considering tympanoplasty, 61.7% had type I with temporalis fascia; 38.3% had type II tympanoplasty (ossiculoplasty) with conchal cartilage augmenting in all (38.3%), except one, who had incus transposition with temporalis fascia alone (Table 4).

**Table 4:** Surgical procedure TFG: Temporalis fascia graft; CCG; conchal cartilage graft, FG: fat graft alone, CI: cartilage interposition, IT: incus transposition *Persistent ear discharge

Surgery Done	No.	TFG	FG	Ossiculoplasty (TFG+ CI) Type II	Ossiculoplasty (TFG+ IT)
Tympanoplasty	76	75 (98.68%)	1 (1.31%)	0	0
Tympanoplasty With Cortical Mastoidectomy*	34	21 (61.76%)	0	12 (35.29 %)	1
Total	110	96	1	12	1

All patients who underwent cortical mastoidectomy had aditus blocked with granulation tissue. In 77%, the perforation’s size was moderate or greater, and 69.2% had discharge for > five years.

**Figure 1:**
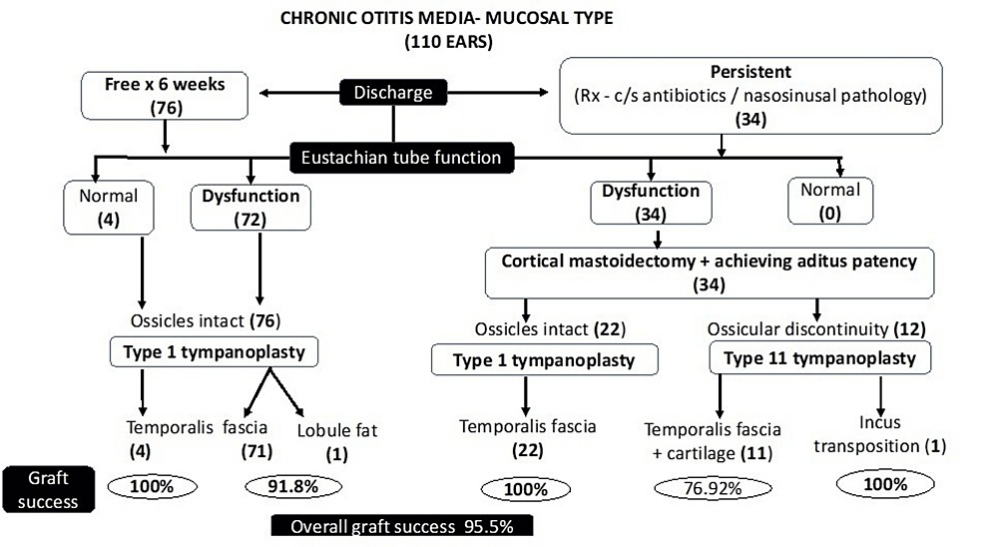
STROBE Rx: Treatment, c/s: culture specific; STROBE: STrengthening the Reporting of OBservational studies in Epidemiology

Graft failure was 4.7% among the 96.4% who had impaired ET function; 4% who had normal ET functions and were also discharge-free had a 100% graft uptake success rate (Figure 1).

An analysis of the number of years of ear discharge with postoperative hearing improvement (to < 25 dB) was conducted. Among those with a duration of ear discharge < one year, 80% had improvement, while those who had discharge from one to five years and > five years improved in only 68% and 63% of patients, respectively, to less than 25 dB. The six-month postoperative air-bone gap (ABG) closure among those with a preoperative ABG of 20-40 dB, 40-50 dB, and >50 dB was found to be statistically significant (P = < 0.02). However, the postoperative hearing threshold became normal only in those with mild to moderate hearing loss (ABG of 20-40 dB), as shown in Table 5.

**Table 5:** AB gap at 6 months postoperatively dB: decibel, AB gap: air-bone gap

Decibel	AB Gap	Mean	N	Standard Deviation	t-Test
20-40 dB	Preoperative	22.8313	83	5.49461	27.43 22.89 p < 0.001
	Postoperative	13.1566	83	4.08890	
40-50 dB	Preoperative	36.0000	16	6.85079	13.54 9.053 p < 0.001
	Postoperative	25.6875	16	7.57820	
>50 dB	Preoperative	44.2727	11	6.88609	9.75 10.077 p < 0.001
	Postoperative	25.4545	11	5.29837	

## Discussion

The incidence of chronic ear disease in India is reported to be 6%, which is significantly higher than that of Western countries (1.8%) [1]. Our prospective study of COM (mucosal type) of 110 ears had ages ranging from 10 to 60 years, the majority being from the 21 to 40 age group with a female predominance of 55%, similar to other reports [5,13], the former [13] being among children.

In our study, the duration of ear discharge by the time the patient sought a specialist’s opinion was more than five years in the majority (49%) of patients, with only 14% having had discharge for less than one year. This was comparable to another report from India, where most patients with inactive mucosal COM (37.69%) had discharge for five to ten years [14].

The most common size of perforation noted in our study was large (52%), followed by moderate (35%), with fewer small and subtotal perforations at 8% and 5%, respectively. In other studies, most COM perforations were reported as moderate (43.75%), followed by subtotal (32.81%), the least being large or small [15]. More recently, studies of two-quadrant perforation have been reported as most common (54.55%), followed by single-quadrant perforations (21.74%), the least being three-quadrant perforations [14].

Our study revealed large and subtotal perforations in 45% of patients with ear discharge for > three years, unlike the 12% with < three years when we analyzed the perforation size and duration of ear discharge. This appeared to be approaching statistical significance and has not been previously reported.

The majority (75%) of our patients had hearing loss ranging from 20 to 40 dB, with 15% and 10% having hearing loss ranging between 40-50 dB and > 50 dB, respectively, consistent with another study [16]. When compared to the perforation size, there was a mean hearing loss of 38 dB and 40 dB in those with large and subtotal perforations, respectively, and those with small or medium perforations had a mean loss of 26 dB and 28 dB, respectively. Studies have reported maximum average hearing loss in the perforations involving all four quadrants, with 44.6 dB in three-quadrant perforations [15] and 51.56 ± 5.1 dB in four-quadrant perforations [14]. A significant increase in hearing loss has been reported with the increase in the size of tympanic membrane perforation (P < 0.0001) [17].

The duration of ear discharge also appears to be related to the mean hearing loss. In our study, when we correlated the number of years of ear discharge with hearing loss, the mean for less than one year was 32.73 dB +/- 12.54 as compared to that of more than five years, which was 38.05 dB +/- 15.10 (Table 3). This is consistent with a recent report where the mean hearing loss of 36.46 ± 8.2 dB, 39.26 ± 5.1 dB, 51.15 ± 7.8 dB, and 52.18 ± 4.2 dB was noted in those with discharge less than one year, one to five years, five to ten years, and more than 10 years, respectively [14]. Our study revealed that 94.5% of patients had conductive and 5.45% had mixed hearing loss, unlike another recent study where conductive, sensorineural, and mixed hearing loss in 67.10%, 16.10%, and 11.80% of patients, respectively, was reported [14].

The modified Toynbee’s inflation and deflation test, which is practical, objective, and suitable, has been recommended for impedance audiometric assessment of ET ventilatory functions prior to tympanoplasty. Our study’s ET ventilation test utilizing the modified Toynbee inflation-deflation test [12,18,19] revealed that 96.4% of patients had ET dysfunction. Previous studies also reported that the Toynbee test revealed ET dysfunction among patients with inactive mucosal COM; 81.8% of patients with central perforation posterior to the malleus handle and 60% among those with subtotal perforation [19,20].

In our study, ossicular discontinuity with erosion of the incus lenticular process was noted in only 11.8%, all having discharge at the time of surgery, which necessitated cortical mastoidectomy. All these patients had antral mucosa hypertrophy and blocked aditus, the majority having moderate to large perforations (90%) and more than five years of ear discharge (75%). Stapes erosion was noted in none. This aligns with a report where none of the patients had stapes involvement [21]. However, that study reported higher percentages of ossicular discontinuity with incus erosion, antral mucosal hypertrophy, and aditus block among those with discharging ears than those with dry ears [21].

Based on the surgical protocol of discharge-free or persistent for less than six weeks, 69% in our study had type I tympanoplasty with temporalis fascia, and 31% had cortical mastoidectomy. In the latter group, the aditus patency was addressed in all, in addition to using conchal cartilage augmented tympanoplasty in all type II (as a single-staged ossiculoplasty) except in one incus transposition (38.3%), while in type I tympanoplasty (61.7%) the temporalis fascia alone was used as graft material. With this tailored approach, our overall graft success was 95.5%. By contrast, 89% and 92% graft success in dry and wet ears, respectively, were reported when cortical mastoidectomy with tympanoplasty was done among all patients with mucosal COM, irrespective of discharge presence [21].

Though the overall graft success rate in our study was 95.5%, it was 100% among the 3.6% who had normal ET functions, and among the remaining 96.4%, graft success was 91.81% despite ET dysfunction. This contradicts two recent prospective studies that reported graft success rates following tympanoplasty in mucosal COM being 87%/70% and 89.49%/50% with normal/impaired ET functions, respectively [15,22].

In our study, analysis of the number of years of ear discharge with postoperative hearing improvement (to < 25 dB) revealed that when the duration of ear discharge was less than one year, 80% improved, while those with one to five years and more than five years discharge only 68% and 63% of patients, respectively, improved (Table 5). Patients with mucosal COM with an increased duration of ear discharge had increased hearing loss with a lesser chance of postoperative improvement to normal levels. This has not been previously reported.

**Figure 2:**
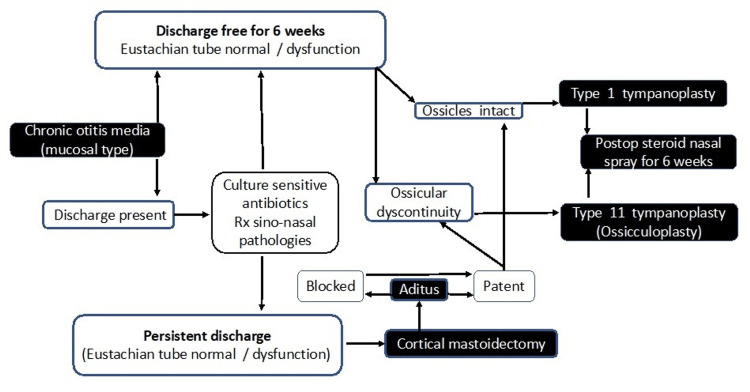
Suggested surgical treatment guidelines for chronic otitis media mucosal disease

Based on our study, we have suggested surgical treatment guidelines for the successful outcome of surgery for mucosal COM (Figure 2).

Limitations

Postoperative follow up was done for six months and not one year as this was a postgraduate thesis protocol and hence time bound. ET functions could have been rechecked following use of steroid nasal sprays postoperatively, after six months.

## Conclusions

In COM, the persistence of ear discharge and its duration (despite culture-specific antibiotics) and ET functions are significant clinical predictors of postoperative graft success and hearing outcomes. The duration of ear discharge is directly proportional to the severity of hearing loss. Patients with more than five years of discharge were noted to have ossicular discontinuity of incudal lenticular process erosion along with three or more quadrant tympanic membrane involvement. The duration of discharge is inversely proportional to postoperative improvement. Early surgical intervention is also indicated in the relatively safe/mucosa COM. Type I tympanoplasty should be done when the patient is discharge-free for over six weeks. Despite addressing the focus of infection in the pharynx and sinonasal region, persistent ear discharge indicates mastoid infection with blocked aditus in addition to ET dysfunction. A cortical mastoidectomy, along with achieving aditus patency, is to be done with tympanoplasty in these patients. Overall graft success of 95.5% is possible despite ET dysfunction in the majority of patients. This is the first study of clinical predictors for postoperative successful hearing outcomes and graft uptake in the chronic mucosal type of OM.
